# Association of preoperative frailty and postoperative delirium with one-year mortality after surgery in older patients with hip fractures

**DOI:** 10.3389/fragi.2026.1923192

**Published:** 2026-10-09

**Authors:** Keqin Zhang, Peidong Zhang, Ling Chen, Housheng Wang, Wubin Chen, Yusheng Zhu, Dai Li, Wenbin Lu

**Affiliations:** 1 Department of Anesthesiology, Yueyang Hospital of Integrated Traditional Chinese and Western Medicine Affiliated to Shanghai University of Traditional Chinese Medicine, Shanghai, China; 2 Department of Medical Imaging, Changhai Hospital, Naval Medical University, Shanghai, China; 3 Faculty of Anesthesiology, Changhai Hospital, Naval Medical University/Second Military Medical University, PLA, Shanghai, China; 4 Department of Anesthesiology and Perioperative Medicine, Shanghai Fourth People’s Hospital, School of Medicine, Tongji University, Shanghai, China; 5 Shanghai Key Laboratory of Anesthesiology and Brain Functional Modulation, Shanghai, China

**Keywords:** elderly, frailty, hip fracture, mortality, postoperative delirium

## Abstract

**Background:**

We aimed to evaluate the incidence of preoperative frailty and postoperative delirium (POD) and the association of preoperative frailty, POD with one-year mortality in older patients undergoing hip fracture surgery.

**Methods:**

Patients with hip fracture were identified from 2018 to 2023 in our hospital. The patients were categorized into four groups: patients without preoperative frailty who did not suffer from POD, patients without preoperative frailty who did suffer from POD, patients with preoperative frailty who did not suffer from POD, and patients with preoperative frailty who did suffer from POD. The primary outcome was one-year mortality after surgery. Multivariate landmark Cox regression analysis was used to assess the associations of preoperative frailty and POD with one-year mortality, and adjusted survival curves from the multivariate Cox model were plotted.

**Results:**

Of 2,659 patients, 1,715 were included in the final analysis. The incidence of preoperative frailty was 24.0% (411/1715), and the POD rate was 16.9% (290/1715). Compared with older patients without preoperative frailty who did not suffer from POD (5.6%, 61/1088), the incidence of one-year mortality was significantly higher in patients without preoperative frailty who did suffer from POD (17.1%, 37/216), patients with preoperative frailty who did not suffer from POD (11.6%, 39/337), and patients with preoperative frailty who did suffer from POD (28.4%, 21/74). Preoperative no-frailty and POD (HR: 2.09, 95% CI: 1.36–3.21), frailty and no-POD (HR: 2.30, 95% CI: 1.51–3.50), and frailty with POD (HR: 3.95, 95% CI: 2.35–6.63) were all independently associated with one-year mortality after surgery in older patients with hip fracture.

**Conclusion:**

Preoperative frailty and POD were each independently associated with increased one-year mortality. Patients with both conditions had the highest observed mortality risk after hip fracture surgery.

## 1 Introduction

Hip fracture is one of the most common and serious injuries in older adults. It poses a major public health challenge with significant impacts on morbidity, mortality, and healthcare costs (Barzilay et al., 2026; Bayar, Cengiz, Erdoğan, Coşkun and Tomak, 2026; Hatano et al., 2026). Despite improvements in surgical techniques and perioperative care, mortality after hip fracture remains high in older patients. One-year mortality rates range from 10% to 30% in this population (García-Tercero et al., 2026; Patel et al., 2026). Therefore, identifying key risk factors for mortality is essential for guiding early intervention, which in turn can improve outcomes in this vulnerable older population.

Frailty is a clinical syndrome characterized by reduced physiological reserves and impaired organ function (Lemieux et al., 2026; Ren et al., 2026; Troy, Ndumele and Dodson, 2026). It makes individuals more susceptible to stressors and less able to recover to their previous state. Growing evidence shows that preoperative frailty is linked to major complications and higher mortality (Ge et al., 2026; Jung, Hong, Jung, Han and Lee, 2026), especially for older patients with hip fracture. In addition, postoperative delirium (POD) is another common and serious complication after hip fracture surgery (Qin et al., 2026; Zhong et al., 2026). It is associated with more postoperative complications, worse functional recovery, and increased mortality (Genda et al., 2026; Han et al., 2026). Moreover, frailty and postoperative delirium are not independent entities but rather share significant pathophysiological overlap and clinical interconnections.

Despite the established individual associations of frailty and POD with poor outcomes after hip fracture surgery (Dwyer et al., 2014; Ferrara et al., 2026), limited research has examined the joint association of these two conditions with long-term mortality. Therefore, the present study was designed to evaluate the incidence of preoperative frailty and POD, and to assess the association of preoperative frailty, postoperative delirium, and their joint association with one-year mortality in older patients undergoing hip fracture surgery.

## 2 Materials and methods

### 2.1 Study population

This retrospective study included hip fracture patients who were admitted to our hospital for elective surgery between 2018 and 2023. Patients aged ≥65 years with radiographically confirmed hip fracture were eligible for inclusion, while those with multiple fractures, pre-existing dementia, tumor, or pre-existing delirium were excluded. The study was approved by the Institutional Review Board in our hospital (CHEC2024-257), which waived the requirement for informed consent due to the retrospective design and use of de-identified data. All procedures were conducted in accordance with the Declaration of Helsinki and reported in accordance with the STROBE guidelines.

### 2.2 Assessment of frailty and delirium after surgery

Preoperative frailty status was assessed using the modified 5-item frailty index (mFI-5) (Klimitz et al., 2026). This index comprises five variables: diabetes mellitus; dependent functional status; history of chronic obstructive pulmonary disease or chronic pneumonia; history of congestive heart failure within 30 days prior to surgery; and history of hypertension requiring pharmacologic therapy. Dependent functional status was defined as requiring assistance from others for activities of daily living-including bathing, feeding, dressing, and ambulation-within 30 days before hip fracture. Each positive item was scored one point, yielding a total score ranging from 0 to 5, with higher scores indicating greater frailty. Consistent with previous studies, the mFI-5 score ≥2 was defined as frailty in this study (Ali et al., 2025; Gupta et al., 2025).

Preoperative delirium was assessed using the Confusion Assessment Method (CAM) in the ward setting. POD was assessed using a collapsed composite outcome that included either a positive delirium screen during the 7-day postoperative period, as determined by the CAM, or chart-based delirium identification (Zarour et al., 2024). The CAM was performed once daily on postoperative days 1–3 by trained anesthesia nurses, covering more than 90% of patients; the remaining patients (including those not followed up within the 3-day window and those during the remaining period up to postoperative day 7) were assessed via CHART-DEL. The latter was defined as documented evidence of acute mental change combined with at least one of several key descriptors following the validated Chart-based Delirium Identification Instrument (CHART-DEL) method (Kaushik et al., 2024; Shenkin et al., 2019; Weiss et al., 2024).

Therefore, CAM alone was used as the sole delirium screening tool in over 90% of patients during the first three postoperative days, whereas CHART-DEL alone was employed in less than 10% of cases. Hypoactive delirium was captured through both CAM (noting lethargy, reduced activity, and withdrawal) and chart review (screening for descriptors such as lethargic, drowsy, and decreased responsiveness).

### 2.3 Data collection and outcome

Based on existing literature and clinical practices, we collected the following variables. The variables included gender, age, body mass index (BMI), American Society of Anesthesiologists (ASA) physical status, and medical history of hypertension, cardiovascular disease, cerebrovascular disease, diabetes, lung disease, kidney disease, and type of fracture, type of surgery, type of anesthesia, time to admission (time from injury to admission), time to surgery (time from admission to surgery), surgery duration, intraoperative blood loss, intraoperative blood transfusion, as well as ICU admission. Laboratory parameters assessed at admission included hemoglobin and albumin. Preoperative hemoglobin and albumin each had 20 missing values, which were handled using multiple imputation.

The primary study endpoint was all-cause mortality at 1 year postoperatively. For patients who died during the hospitalization, mortality information was obtained from hospital medical records. For patients discharged alive, follow-up was conducted via structured telephone interviews after surgery. All patients or family members were interviewed by trained research assistants to confirm whether the patient had died and, if so, the exact date of death. There were no losses to follow-up in our cohort due to a follow-up of a single survival status.

### 2.4 Statistical analysis

According to baseline frailty status and POD, patients were categorized into four groups: patients without preoperative frailty who did not suffer from POD, patients without preoperative frailty who did suffer from POD, patients with preoperative frailty who did not suffer from POD, and patients with preoperative frailty who did suffer from POD. Data are summarized as mean ± standard deviation for normally distributed continuous variables, analyzed with the one-way ANOVA; or as median and interquartile range for skewed data, compared with the Kruskal–Wallis test. Categorical variables are expressed as number (percentage) and were evaluated using the chi-square test.

To avoid immortal time bias, we performed a landmark Cox regression analysis to evaluate the independent association of preoperative frailty and postoperative delirium with one-year mortality after surgery. We also tested the interaction between frailty and POD by including a frailty × POD product term in this model. The proportional hazards assumption was assessed using Schoenfeld residuals, with P > 0.05 considered to indicate that the assumption was not violated.

Based on the observed distribution of POD onset in our cohort and the fact that nearly 90% of all POD events occurred within the first three postoperative days (Supplementary Figure S1), we prespecified postoperative day 3 as the landmark time point. No patient in our cohort died within the first 3 days; therefore, all patients were included in the landmark analysis. In this landmark analysis, postoperative day 3 was used as the time origin, and POD exposure was defined as the occurrence of delirium within the first three postoperative days. The cumulative survival probabilities of these groups were estimated via adjusted survival analysis from the multivariate Cox model.

Covariates for the multivariate Cox regression models were selected based on clinical relevance, excluding the components of the mFI-5. Model I adjusted for nothing. Model II adjusted for age, gender, and BMI. Model III adjusted for model II plus ASA physical status, kidney disease, cerebrovascular disease, type of fracture, type of surgery, type of anesthesia, time to admission, time to surgery, surgery duration, intraoperative blood loss, intraoperative blood transfusion, ICU admission, hemoglobin, and albumin. All analyses were performed using R software (version 4.2.2, The R Foundation) and Free Statistics version 1.7.1. A two-sided p-value < 0.05 was deemed statistically significant.

## 3 Results

A total of 2,659 patients who underwent hip fracture surgery were identified. After excluding 944 patients for not meeting the inclusion criteria (due to multiple fractures, dementia, tumor, delirium or aged less than 65 years), the final analysis comprised 1,715 individuals. The enrollment process is detailed in Figure 1. The distribution of POD onset by postoperative day is presented in Supplementary Figure S1, with the majority of events occurring within the first 3 days after surgery.

**FIGURE 1:**
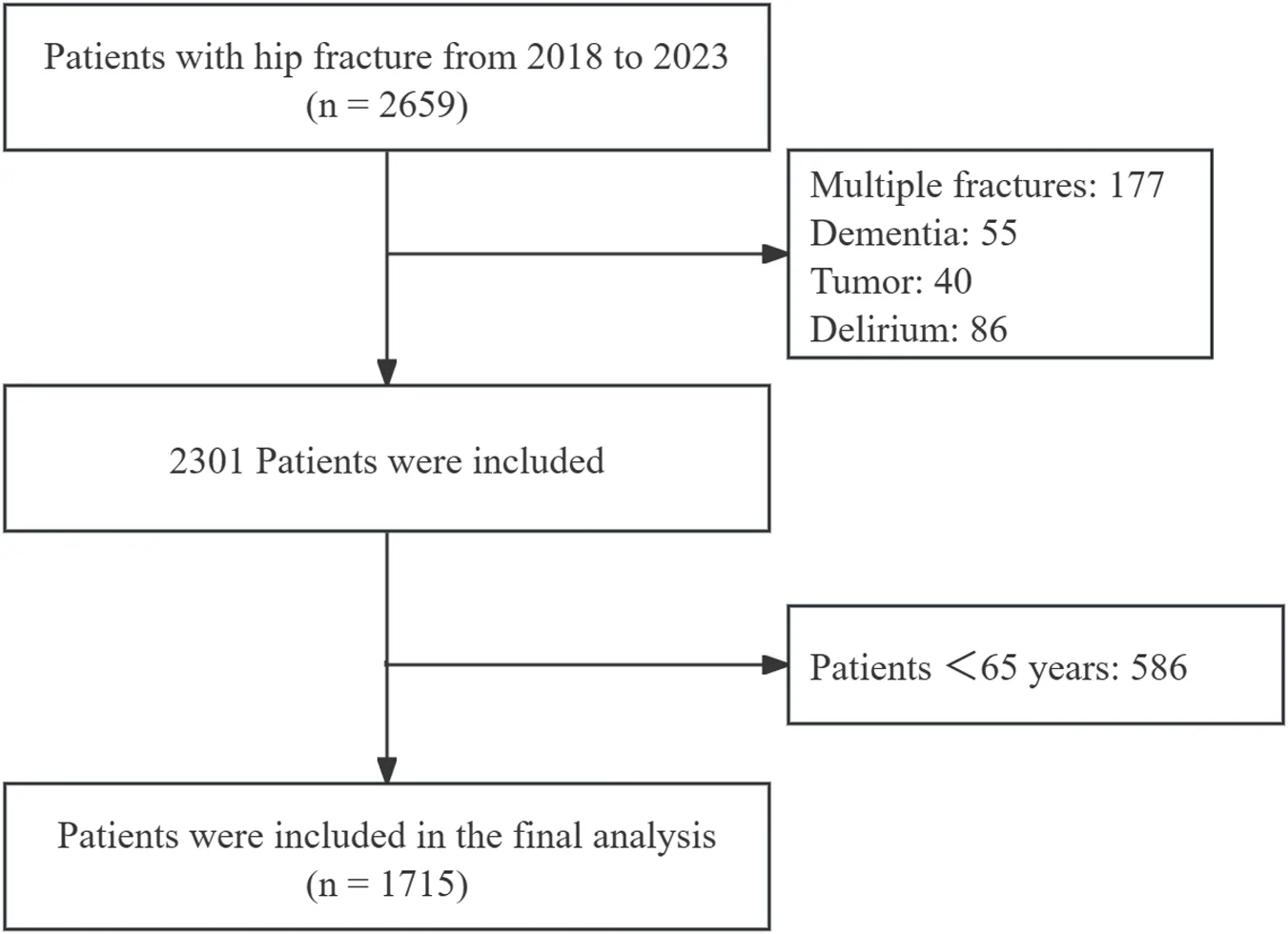
Flowchart of the study population selection process.

We identified patients without preoperative frailty who did not suffer from POD (63.4%), without preoperative frailty who did suffer from POD (12.6%), with preoperative frailty who did not suffer from POD (19.7%), or with preoperative frailty who did suffer from POD (4.3%). Baseline characteristics were compared between older patients included in the four groups (Table 1). The median time from injury to hospital admission was 19.0 h (6.0–48.0), and the median time from hospital admission to surgery was 69.6 h (44.9–99.0). Compared with patients without preoperative frailty who did not suffer from POD, patients with preoperative frailty who did suffer from POD were more likely to have advanced age, higher BMI, ASA physical status ≥ III, ICU admission, and comorbidities including cerebrovascular disease, kidney disease, and lower hemoglobin and albumin levels (all *P* < 0.05).

**TABLE 1:** Baseline information between different groups based preoperative frailty and POD.

Variables	No frailty and no POD (n = 1,088)	No frailty and POD (n = 216)	Frailty and no POD (n = 337)	Frailty and POD (n = 74)	*P*
Age (years)	78.8 ± 8.2	84.5 ± 7.4	78.5 ± 7.3	82.5 ± 7.6	<0.001
Gender	​	​	​	​	0.258
Female	731 (67.2)	159 (73.6)	236 (70.0)	49 (66.2)	​
Male	357 (32.8)	57 (26.4)	101 (30.0)	25 (33.8)	​
BMI (kg/m^2^)	22.2 ± 3.7	21.9 ± 3.6	23.4 ± 3.7	22.9 ± 3.7	<0.001
ASA physical status	​	​	​	​	<0.001
< III	712 (65.4)	116 (53.7)	172 (51.0)	34 (45.9)	​
≥III	376 (34.6)	100 (46.3)	165 (49.0)	40 (54.1)	​
Hypertension	451 (41.5)	101 (46.8)	324 (96.1)	70 (94.6)	<0.001
Diabetes	106 (9.7)	21 (9.7)	292 (86.6)	67 (90.5)	<0.001
Cardiovascular disease	224 (20.6)	46 (21.3)	103 (30.6)	28 (37.8)	<0.001
Cerebrovascular disease	137 (12.6)	34 (15.7)	79 (23.4)	19 (25.7)	<0.001
Kidney disease	27 (2.5)	5 (2.3)	16 (4.7)	7 (9.5)	0.006
Lung disease	40 (3.7)	12 (5.6)	67 (19.9)	21 (28.4)	<0.001
Type of fracture	​	​	​	​	0.960
Femoral neck fracture	579 (53.2)	114 (52.8)	175 (51.9)	36 (48.6)	​
Intertrochanteric femoral fracture	482 (44.3)	98 (45.4)	156 (46.3)	37 (50.0)	​
Subtrochanteric femoral fracture	27 (2.5)	4 (1.9)	6 (1.8)	1 (1.4)	​
Type of surgery	​	​	​	​	0.137
Hemiarthroplasty	321 (29.5)	80 (37.0)	119 (35.3)	24 (32.4)	​
Total hip arthroplasty	110 (10.1)	22 (10.2)	21 (6.2)	7 (9.5)	​
Intramedullary nailing	388 (35.7)	75 (34.7)	115 (34.1)	23 (31.1)	​
Screw fixation	269 (24.7)	39 (18.1)	82 (24.3)	20 (27.0)	​
Type of anesthesia	​	​	​	​	0.166
General anesthesia	156 (14.3)	44 (20.4)	53 (15.7)	12 (16.2)	​
Spinal anesthesia	932 (85.7)	172 (79.6)	284 (84.3)	62 (83.8)	​
Time to admission (h)	17.0 (6.0, 34.0)	24.0 (10.0, 72.0)	20.0 (6.5, 50.0)	13.8 (5.2, 47.9)	<0.001
Time to surgery (h)	67.2 (44.3, 98.3)	68.1 (43.4, 96.2)	70.6 (47.4, 99.4)	73.3 (57.5, 126.1)	0.023
Surgery duration (min)	100.0 (85.0, 125.0)	100.0 (80.0, 120.0)	100.0 (85.0, 130.0)	100.0 (80.0, 120.8)	0.374
Intraoperative blood loss (mL)	200.0 (100.0, 200.0)	200.0 (100.0, 200.0)	150.0 (100.0, 200.0)	150.0 (100.0, 300.0)	0.927
Intraoperative blood transfusion	452 (41.5)	106 (49.1)	149 (44.2)	38 (51.4)	0.096
ICU admission	120 (11.0)	26 (12.0)	49 (14.5)	18 (24.3)	0.005
Hemoglobin (g/L)	116.0 (102.0, 129.0)	112.0 (94.8, 125.0)	112.0 (99.0, 126.0)	115.0 (93.5, 125.0)	0.008
Albumin (g/L)	36.0 (33.0, 39.0)	34.0 (32.0, 38.0)	36.0 (33.0, 39.0)	35.0 (32.2, 38.0)	<0.001
One-year mortality	61 (5.6)	37 (17.1)	39 (11.6)	21 (28.4)	<0.001

Data are presented as mean ± SD, or median (IQR) and frequency (%). BMI, body mass index; ASA, american society of anesthesiologists; ICU, intensive care unit; POD, postoperative delirium. Hypertension, diabetes, pulmonary disease, and heart failure (from cardiovascular disease) are among the components of the mFI-5.

In addition, compared with patients without preoperative frailty who did not suffer from POD (5.6%, 61/1,088), the incidence of one-year mortality was significantly higher in patients without preoperative frailty who did suffer from POD (17.1%, 37/216), patients with preoperative frailty who did not suffer from POD (11.6%, 39/337), and patients with preoperative frailty who did suffer from POD (28.4%, 21/74) (*P* < 0.001 for all comparisons).

The Schoenfeld residual analysis revealed that the primary exposure variable, covariates, and the global Schoenfeld residuals showed no significant correlation with time (all *P* > 0.05), indicating that the proportional hazards assumption was satisfied for the Cox regression model (Supplementary Table S1). Table 2 summarizes the adjusted hazard ratio (HR) for one-year mortality among older patients in four groups. Preoperative no-frailty and POD (adjusted HR: 2.09, 95% CI: 1.36–3.21; adjusted *P*-value = 0.001), frailty and no-POD (adjusted HR: 2.30, 95% CI: 1.51–3.50; adjusted *P*-value < 0.001), and frailty with POD (adjusted HR: 3.95, 95% CI: 2.35–6.63; adjusted *P*-value < 0.001) were all independently associated with one-year mortality after surgery in older patients with hip fracture. However, the interaction term for frailty and POD was not statistically significant when added to the multivariate Cox regression model (*P* > 0.05).

**TABLE 2:** Univariate and multivariate landmark Cox regression analysis to assess the association of properative frailty and POD with one-year mortality in the elderly patients with hip fracture surgery.

Variable	Model I		Model II		Model III	
	HR (95% CI)	*P*	HR (95% CI)	*P*	HR (95% CI)	*P*
No-frailty and no-POD	Rf	​	Rf	​	Rf	​
No-frailty and POD	3.29 (2.18∼4.95)	<0.001	2.11 (1.39∼3.19)	<0.001	2.09 (1.36∼3.21)	0.001
Frailty and no-POD	2.12 (1.42∼3.18)	<0.001	2.57 (1.71∼3.86)	<0.001	2.30 (1.51∼3.50)	<0.001
Frailty and POD	5.94 (3.62∼9.76)	<0.001	4.76 (2.88∼7.85)	<0.001	3.95 (2.35∼6.63)	<0.001
Interaction (frailty × POD)	1.30 (0.65∼2.59)	0.453	1.33 (0.67∼2.67)	0.410	1.17 (0.58∼2.37)	0.658

Model I adjusted for nothing. Model II, adjusted for age, gender, and BMI., Model III, adjusted for model II, plus, ASA, physical status, kidney disease, cerebrovascular disease, type of fracture, type of surgery, type of anesthesia, time to admission, time to surgery, surgery duration, intraoperative blood loss, intraoperative blood transfusion, ICU, admission, hemoglobin, and albumin; POD, postoperartive delirium; BMI, body mass index; ASA, american society of anesthesiologists; ICU, intensive care unit; HR, hazard ratio; CI, confidence interval; Rf, reference.

We also conducted a sensitivity analysis limited to patients who underwent delirium assessment using CAM alone, and the results were consistent with the primary analysis (Supplementary Table S2). Additionally, a multivariate Cox regression model with frailty and POD entered simultaneously as two separate binary variables revealed that both preoperative frailty (HR: 1.61, 95% CI: 1.13–2.30) and POD (HR: 1.95, 95% CI: 1.38–2.75) were independently associated with one-year postoperative mortality (Supplementary Table S3).

Adjusted survival curves also indicated that patients with preoperative frailty who suffered from POD, without preoperative frailty who did suffer from POD, or with preoperative frailty who did not suffer from POD had a lower one-year survival rate than that in patients without preoperative frailty who did not suffer from POD (*P* < 0.001) (Figure 2).

**FIGURE 2:**
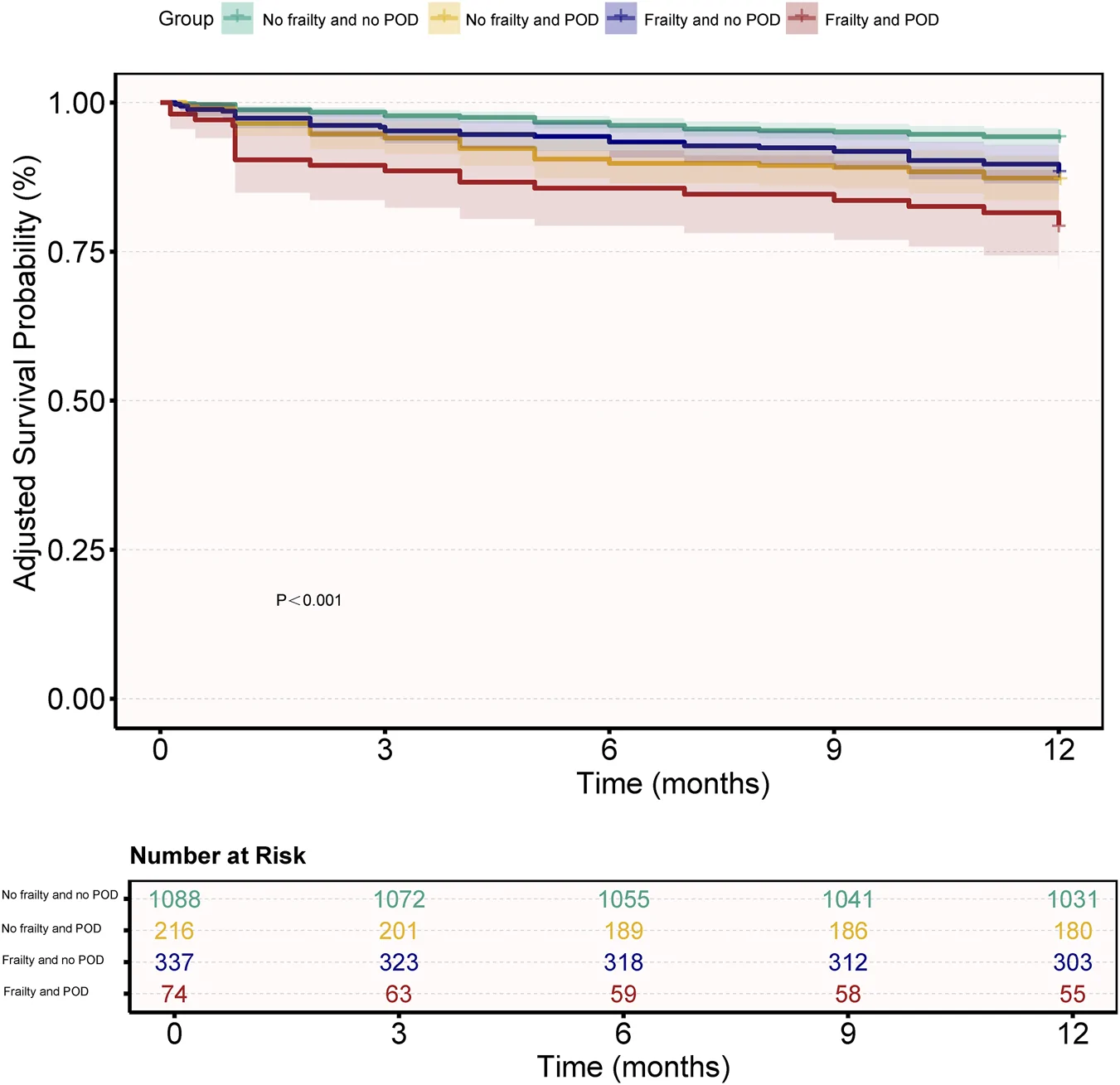
Adjusted survival curves of one-year mortality after surgery based on preoperative frailty and POD in older patients with hip fracture.

## 4 Discussion

In this retrospective study of 1,715 older patients undergoing hip fracture surgery, we demonstrated that preoperative frailty and delirium after surgery were each independently associated with increased one-year mortality. Notably, the coexistence of both conditions conferred a substantially higher risk of mortality, with the one-year mortality rate reaching 28.4% (21/74) in patients with both frailty and POD compared to only 5.6% (61/1,088) in those with neither condition.

Our results regarding the association between preoperative frailty and mortality are consistent with previous investigations demonstrating that frailty serves as a robust predictor of adverse outcomes following surgeries. Multiple studies have identified frailty as an independent risk factor for complications, functional decline, and mortality in geriatric trauma patients (Abdelrahman et al., 2025; Al Ma’ani et al., 2025; Hamdioğlu, Altuntaş, Çelik and Yavaşi, 2025). Our findings further reinforce this evidence, showing that preoperative frailty was significantly associated with increased long-term postoperative mortality risk even after adjusting for the potential confounding factors.

Similarly, the observed relationship between POD and increased mortality aligns with extensive literature documenting delirium as a marker of heightened vulnerability and poorer long-term prognosis. Previous studies have demonstrated that the occurrence of delirium not only prolongs hospital stays and increases healthcare costs (Kishi et al., 2026) but also is independently associated with persistent postoperative cognitive decline and elevated mortality (Huang et al., 2021; Umoh et al., 2026; Mohammad et al., 2026). Our data further support this view and suggest that POD was associated with increased one-year mortality after surgery in older patients with hip fractures.

However, unlike many previous studies that examined these conditions in isolation, our study specifically quantified the joint association of frailty and POD with mortality. While individual studies have reported mortality rates ranging from 10% to 30% following hip fracture (Harvey et al., 2024), our analysis revealed considerable heterogeneity within this population, with mortality varying fivefold depending on the presence or absence of these two risk factors, indicating a joint association of frailty and POD with substantially increased mortality risk.

Frailty represents a state of diminished physiological reserve and dysregulated homeostatic mechanisms, characterized by chronic low-grade inflammation, sarcopenia, and immunosenescence (Güven, 2026; Gao and Zhang, 2026). Preoperative frailty further predisposes patients to postoperative stress, inflammation, and metabolic disturbances, thereby increasing the risk of postoperative delirium (Chen, Fan, Tu and Chen, 2024; Wiredu, Qu, Turco, McKay and Akeju, 2025). The co-occurrence of frailty and POD confers a disproportionately elevated risk of postoperative mortality. However, further research is needed to demonstrate the specific underlying mechanisms and provide integrated intervention strategies to improve long-term survival in this vulnerable population.

The present study possesses several strengths, including its relatively large sample size, and comprehensive adjustment for potential confounders. Furthermore, this study demonstrates that the joint association of preoperative frailty and POD with increased one-year mortality following surgery. However, several limitations should be acknowledged. The retrospective design precludes causal inference and may be susceptible to selection bias and unmeasured confounding, such as perioperative complications or postoperative functional status. In addition, delirium assessment relied partly on chart review methods, which may underestimate true incidence compared to prospective daily screening, and formal cognitive assessment at baseline was not performed in our cohort. Meanwhile, the mFI-5, while practical, captures only a limited dimension of the multidimensional frailty construct. Moreover, although landmark analysis was applied, residual time-dependent bias may still be present. Finally, the single-center nature of the study may limit generalizability to other healthcare settings with different patient demographics or perioperative protocols.

## 5 Conclusion

In conclusion, preoperative frailty and postoperative delirium were each independently associated with one-year mortality following hip fracture surgery. Patients with both conditions had the highest observed mortality risk after hip fracture surgery. Future research should develop targeted interventions to counter their effects on patient outcomes.

## Data Availability

The original contributions presented in the study are included in the article/Supplementary Material, further inquiries can be directed to the corresponding authors.
