# Supplementary material for: Association of preoperative frailty and postoperative delirium with one-year mortality after surgery in older patients with hip fractures
Source: Front Aging. 2026 Oct 9;7:1923192. doi: 10.3389/fragi.2026.1923192 (PMC13690787; doi:10.3389/fragi.2026.1923192)
Supplement: Supplementary file 1 [file DataSheet1.docx]

Supplementary Material

Association of preoperative frailty and postoperative delirium with one-year mortality after surgery in older patients with hip fractures

Keqin Zhang ^1, *^, Peidong Zhang ^2, *^, Ling Chen ^3, *^, Housheng Wang ^3^, Wubin Chen ^3^, Yusheng Zhu ^4,5 #^, Dai Li ^3, #^, Wenbin Lu ^3, #^

^1^ Department of Anesthesiology, Yueyang Hospital of Integrated Traditional Chinese and Western Medicine Affiliated to Shanghai University of Traditional Chinese Medicine, Shanghai, 200437, China

^2^ Department of medical imaging, Changhai Hospital, Naval Medical University, Shanghai, 200433, China

^3^ Faculty of Anesthesiology, Changhai Hospital, Naval Medical University/Second Military Medical University, PLA, Shanghai, 200433, China

^4^ Department of Anesthesiology and Perioperative Medicine, Shanghai Fourth People’s Hospital, School of Medicine, Tongji University, Shanghai, 200434, China

^5^ Shanghai Key Laboratory of Anesthesiology and Brain Functional Modulation, Shanghai, 200434, China

* Equal Contribution

\# Corresponding author

Correspondence:

Wenbin Lu, M.D., Ph.D.

Faculty of Anesthesiology,

Changhai Hospital, Second Military Medical University,

168, Changhai Road, 200433, Shanghai, China.

Tel: +86-021-31161841

Fax: +86-021-31161841

E-mail: chwenbinlu@sina.com

Supplementary Figure 1 The distribution of POD onset by postoperative day

Supplementary Table 1 Schoenfeld residual tests for the proportional hazards assumption in the Cox regression model.

| Variable | Chi-square | Df | P |
| --- | --- | --- | --- |
| Age | 3.653 | 1 | 0.056 |
| Gender | 0.286 | 1 | 0.593 |
| BMI | 0.010 | 1 | 0.919 |
| ASA physical status | 1.494 | 1 | 0.222 |
| Cerebrovascular disease | 0.736 | 1 | 0.391 |
| Kidney disease | 0.172 | 1 | 0.678 |
| Type of surgery | 3.016 | 3 | 0.389 |
| Type of fracture | 0.204 | 2 | 0.903 |
| Type of anesthesia | 0.453 | 1 | 0.501 |
| Time to admission | 0.121 | 1 | 0.728 |
| Time to surgery | 3.011 | 1 | 0.083 |
| Surgery duration | 3.594 | 1 | 0.058 |
| Intraoperative blood loss | 0.784 | 1 | 0.376 |
| Intraoperative blood transfusion | 0.442 | 1 | 0.506 |
| ICU admission | 0.393 | 1 | 0.531 |
| Preoperative frailty and POD | 6.001 | 3 | 0.112 |
| Albumin | 0.268 | 1 | 0.605 |
| Hemoglobin | 0.198 | 1 | 0.656 |
| Global | 24.929 | 23 | 0.355 |

BMI, body mass index; ASA, American Society of Anesthesiologists; ICU, intensive care unit. POD, postoperative delirium. Df, degrees of freedom.

Supplementary Table 2 Sensitivity analysis to assess the association of properative frailty and POD with one-year mortality in 1565 elderly patients with hip fracture surgery using CAM.

| **Variable** | **Model I** | |  | **Model II** | |  | **Model III** | |
| --- | --- | --- | --- | --- | --- | --- | --- | --- |
|  | **HR (95% CI)** | ***P*** |  | **HR (95% CI)** | ***P*** |  | **HR (95% CI)** | ***P*** |
| No-frailty and no-POD | Rf |  |  | Rf |  |  | Rf |  |
| No-frailty and POD | 2.91 (1.94~4.39) | <0.001 |  | 1.85 (1.22~2.80) | 0.004 |  | 1.85 (1.21~2.85) | 0.005 |
| Frailty and no-POD | 2.08 (1.39~3.11) | <0.001 |  | 2.40 (1.59~3.60) | <0.001 |  | 2.13 (1.40~3.25) | <0.001 |
| Frailty and POD | 5.27 (3.21~8.65) | <0.001 |  | 4.18 (2.53~6.89) | <0.001 |  | 3.51 (2.09~5.89) | <0.001 |
| Interaction (frailty × POD) | 1.33 (0.67~2.64) | 0.421 |  | 1.43 (0.72~2.86) | 0.309 |  | 1.32 (0.65~2.67) | 0.443 |

Model I adjusted for nothing. Model II adjusted for age, gender, and BMI. Model III adjusted for model II plus ASA physical status, kidney disease, cerebrovascular disease, type of fracture, type of surgery, type of anesthesia, time to admission, time to surgery, sugery duration, intraoperative blood loss, intraoperative blood transfusion, ICU admission, hemoglobin, and albumin. POD, postoperartive delirium; BMI, body mass index; ASA, American Society of Anesthesiologists; ICU, intensive care unit. CAM, confusion assessment method; HR, hazard ratio; CI, conﬁdence interval; Rf, reference.

Supplementary Table 3 Multivariable Cox regression analysis for one-year mortality in the elderly patients with hip fracture surgery.

| Variable | HR (95% CI) | P |
| --- | --- | --- |
| POD | 1.95 (1.38~2.75) | <0.001 |
| Frailty | 1.61 (1.13~2.30) | 0.008 |
| Age | 1.09 (1.06~1.12) | <0.001 |
| Male | 1.80 (1.28~2.52) | 0.001 |
| BMI | 0.97 (0.93~1.02) | 0.205 |
| ASA≥III | 1.07 (0.77~1.49) | 0.674 |
| Cerebrovascular | 1.51 (1.03~2.23) | 0.036 |
| Kidney disease | 2.21 (1.23~3.97) | 0.008 |
| Total hip arthroplasty | 0.36 (0.09~1.52) | 0.165 |
| Intramedullary nailing | 1.16 (0.60~2.25) | 0.66 |
| Screw fixation | 1.55 (0.85~2.82) | 0.155 |
| Intertrochanteric femoral fracture | 0.81 (0.45~1.46) | 0.478 |
| Subtrochanteric femoral fracture | 0.50 (0.14~1.87) | 0.307 |
| Spinal anesthesia | 0.75 (0.49~1.16) | 0.195 |
| Surgery duration | 1 (0.99~1) | 0.108 |
| Intraoperative blood loss | 1 (1~1) | 0.205 |
| Intraoperative blood transfusion | 1.36 (0.94~1.96) | 0.105 |
| Time to admission | 1 (1~1) | 0.39 |
| Time to surgery | 0.98 (0.91~1.05) | 0.578 |
| ICU admission | 1.25 (0.77~2.03) | 0.370 |
| Albumin | 0.95 (0.91~0.99) | 0.011 |
| Hemoglobin | 0.99 (0.98~1) | 0.247 |

POD, postoperartive delirium; BMI, body mass index; ASA, American Society of Anesthesiologists; ICU, intensive care unit. HR, hazard ratio; CI, conﬁdence interval.
